# The Hennepin Healthcare Forced Ketamine Studies, Excited Delirium, and Police Violence

**DOI:** 10.1002/hast.4985

**Published:** 2025-10-15

**Authors:** Carl Elliott, Lauren Wilson

**Keywords:** ketamine adverse effects, chemical restraint, emergency medical services, ethics, research ethics, informed consent, vulnerable populations

## Abstract

*In the summer of 2018, the* Minneapolis Star Tribune *reported clinical trials at Hennepin County Medical Center in which emergency medical personnel were injecting agitated individuals with ketamine, often at the urging of police. These individuals were enrolled in the trials without their knowledge or consent. In one of the studies, nearly 40 percent of subjects given ketamine experienced breathing issues so serious that they had to be intubated. Many subjects were members of vulnerable, marginalized groups. In this paper, we describe the ways in which the Hennepin Healthcare ketamine studies violated federal research guidelines. We consider the troubling relationship between Hennepin Healthcare and law enforcement, as well as the concept of* excited delirium. *Finally, we consider some alternative ways of conceptualizing clinical trials in which the intervention may not benefit subjects. We compare the ketamine trials to clinical trials of chemical restraints in nursing homes and other health care institutions and also to studies of “nonlethal” weapons*.

## Article

Brittany Buckley was asleep on the couch when the police arrived. It was the anniversary of her father's death, and she had been drinking all day.^1^ A concerned friend had called 911 and asked for a welfare check. After a few minutes of conversation, the police called for an ambulance, and paramedics from Hennepin Healthcare told Buckley she was being taken to the hospital. When Buckley objected, she was handcuffed, carried to the ambulance, and strapped to a gurney. Then paramedics injected her with 150 mg of ketamine, a dissociative anesthetic. Within minutes, Buckley lost consciousness and began struggling to breathe.

When Buckley regained consciousness a day later, she found herself intubated and restrained on a hospital bed. Next to her bed was a document titled “Notification of Enrollment.” “You are receiving this form because you or someone you care for was included in a research study examining patients with agitation,” the document said. The study compared two drugs, ketamine and midazolam. “This is all I got,” Buckley told the *Minneapolis* *Star Tribune*. “Just this form saying that I'm part of their little test.”^2^

Buckley's experience was only part of a series of alarming revelations about medical research at Hennepin Healthcare reported by Andy Mannix of the *Star Tribune* in the summer of 2018.^3^ The revelations were initially triggered by a leaked report from the Office of Police Conduct Review.^4^ That report revealed that Hennepin Healthcare medical responders had drugged people with ketamine at the direction of Minneapolis police officers, often forcibly in situations where it did not appear necessary. On several occasions, police officers held people down as medical responders gave the shot. One person injected with ketamine was a suspected jaywalker. Others were already handcuffed or strapped to a stretcher when they were injected. In some cases, the ketamine resulted in cardiac or respiratory failure. A woman who was maced by the police asked for asthma medication. Instead, paramedics handcuffed her to a stretcher and gave her ketamine. Her breathing stopped, and she had to be resuscitated at the hospital.

A week after the *Star Tribune*'s initial report came an unsettling update: the incidents involving ketamine and the police were part of two research studies at Hennepin County Medical Center, a safety‐net hospital in downtown Minneapolis and the flagship medical center for Hennepin Healthcare.^5^ The stated purpose of the studies was to test how safe and effective ketamine was in managing agitation in prehospital settings, usually in situations where emergency responders have been called. But in neither study did the emergency responders obtain informed consent from the subjects, nor did they attempt to get consent from a surrogate. In fact, in several of the cases reported by the Office of Police Conduct Review, the subjects begged not to be injected with ketamine.

Ketamine is not approved by the U.S. Food and Drug Administration to manage agitation, much less to be administered by paramedics or emergency medical technicians. The labeling warns that the drug should be used “by or under the direction of physicians experienced in administering general anesthetics and in maintenance of an airway and in the control of respiration.”^6^ In clinical settings, ketamine is used mainly for procedural sedation and for general anesthesia in combination with other agents. Known as a “dissociative” anesthetic, ketamine produces a sense of bodily detachment and, at higher doses, can cause hallucinations. Over the past decade, some clinicians have begun to prescribe ketamine off‐label as a therapy for treatment‐resistant depression. Psychiatric researchers have also used it in psychosis challenge studies to mimic the experience of schizophrenia. Because it is a colorless, fast‐acting agent that can produce confusion and amnesia, ketamine is sometimes used as a date‐rape drug.

The *Star Tribune*'s revelations touched off a heated public controversy. In a letter signed by over sixty bioethicists, physicians, and other academics, Public Citizen asked the FDA and the Office for Human Research Protection to investigate possible violations of federal research regulations. The *Star Tribune* editorial board called for an independent probe. Yet Hennepin Healthcare officials defended the studies vigorously, and local emergency medical services (EMS) medical directors claimed that ketamine was safe and well studied. At the heart of the EMS medical directors’ defense was the claim that ketamine was the best way to treat or prevent “excited delirium,” a life‐threatening condition that must be treated rapidly with sedation. The EMS medical directors asserted, “When left unchecked and untreated, the outcome is often death.”^7^

To many in the local community, the most inflammatory aspect of the ketamine studies was the involvement of the police. While the murder of George Floyd was still two years away, relations between local police and the Black community in 2018 were already tense. Over the previous few years, Minneapolis police had killed several unarmed Black men, including, in 2015, Jamar Clark, whose death led to widespread protests and an eighteen‐day occupation of a North Minneapolis police precinct.^8^ For this reason, few members of the Black community were surprised when the Office of Police Conduct Review reported that 40 percent of the people injected with ketamine were Black and 10 percent were American Indian.^9^ The population of Hennepin County is 14.5 percent Black and 1.1 percent American Indian.

The controversy was not limited to Hennepin Healthcare. Shortly after the *Star Tribune*'s revelations about the Hennepin Healthcare studies, John Powell, a forty‐eight‐year‐old Black man, filed a lawsuit claiming that, in 2015, Minneapolis police officers had mistaken him for another person and directed paramedics at North Memorial Hospital to sedate him with ketamine, sending him to the intensive care unit.^10^ In August 2020, Joe Baker, a paramedic in Woodbury, a suburb of St. Paul, filed a whistleblower lawsuit claiming that police had pressured him to administer ketamine during an arrest.^11^ Two years later, Brandon Currie, a thirty‐five‐year‐old man in Elk River, Minnesota, filed a lawsuit after police directed paramedics to inject him with ketamine and his kidneys shut down.^12^

Over six years after the Hennepin ketamine studies were revealed, their legacy is still contested. The FDA found serious safety violations and sent warning letters to two investigators.^13^ Among the potential violations noted in the FDA inspection of the Hennepin Healthcare institutional review board (IRB) were the failure to obtain informed consent and a failure to safeguard the welfare of vulnerable subjects.^14^ Yet three investigations funded by Hennepin Healthcare itself exonerated the organization of blame.^15^ A lawsuit filed by Brittany Buckley was dismissed on grounds of qualified immunity; the court found that Buckley had failed to show that her rights had been violated under existing law.^16^ Hennepin Healthcare has not apologized for the studies, nor has there been any public indication that they have contacted subjects to notify them of the FDA findings.^17^

What has changed dramatically since 2018 is the political landscape surrounding police violence. The most momentous shift, of course, was the death of George Floyd at the hands of the Minneapolis police in 2020, an event that triggered widespread protests and a national debate over policing and racism. In Minneapolis, the debate has been further complicated by revelations about a troubling relationship between the police and Hennepin Healthcare. Not only were police directing Hennepin Healthcare emergency responders to sedate agitated individuals with ketamine, but two of the clinical investigators on the ketamine studies were themselves licensed police officers.^18^ In addition, Hennepin Healthcare and many of its physicians had long‐standing financial and professional relationships with Axon, the manufacturer of Taser stun guns.^19^ Some of these Axon‐funded physicians were responsible for training police officers in how to respond to individuals who appeared to be delirious or mentally ill.

Even more significant has been the collapse of the diagnosis of “excited delirium,” a medical condition supposedly characterized by extreme agitation, violence, inappropriate behavior, and feats of superhuman strength. In 2018, it was still plausible (if controversial) to cite the grave risks of excited delirium as a justification for forced sedation with ketamine. Today, however, excited delirium is widely seen as a pseudodiagnosis used to explain away deaths that have occurred at the hands of the police.^20^ No major medical organization supports its legitimacy.^21^

As a result, the Hennepin Healthcare ketamine studies look much different than they did in 2018. When the FDA investigated the ketamine studies, it treated them as conventional, later‐stage clinical trials: studies of investigational treatments for a potentially dangerous medical condition. Its investigation made no mention of law enforcement, much less excited delirium. Yet a full evaluation of the ethics of the ketamine studies demands an understanding of the larger political context in which they were conducted. What are we to make of medical research in which some of the physician‐investigators are licensed police officers, where the study drugs may be administered at the direction of the police, and for which one of the main justifications has been unmasked as a rationale for police violence?

In this article, we attempt to answer that question. We begin with a summary of the most important ways in which the Hennepin ketamine studies violated federal research guidelines. Next, we consider the complex relationship between Hennepin Healthcare and law enforcement organizations, including the financial and professional conflicts of interest such relationships raise. We also present a brief history of the concept of excited delirium, explaining why its collapse is important for understanding the ethics of the ketamine studies. Finally, we consider some alternative ways of conceptualizing the intervention being tested in the ketamine studies. We compare the ketamine studies to studies of “chemical restraints” in hospitals, nursing homes, and other health care institutions and also to studies of “nonlethal” or “less lethal” weapons deployed by the police.

## Waiver of Informed Consent and “Minimal Risk”

Both of the controversial Hennepin Healthcare ketamine studies involved paramedics responding to calls involving agitated individuals, many of whom were experiencing a mental health crisis or the effects of drugs or alcohol. The aim of the studies was to compare different ways of sedating such individuals quickly and safely. The first study, published in 2016, enrolled 146 subjects who were “severely” agitated, a state that was defined as loud and very anxious but not violent or combative. The study compared intramuscular injections of ketamine (Ketalar) to haloperidol (Haldol), an antipsychotic drug.^22^ The primary outcome measure for the study was “time to adequate sedation” as judged by the paramedics, who carried stopwatches. By this measure, ketamine proved much better. The median time to adequate sedation for the ketamine group was a mere five minutes, compared to seventeen minutes for those sedated with haloperidol.

Yet nearly 40 percent of subjects given ketamine experienced breathing issues so serious that they had to be intubated. That was almost ten times the intubation rate of subjects given haloperidol. In a second analysis that included patients judged to be “profoundly” agitated, 57 percent of patients given ketamine required intubation^23^—a rate so high that a *NEJM Journal Watch* reviewer called it “astonishing” and “not acceptable.”^24^ Left unmentioned was the fact that ketamine can cause amnesia, leaving some subjects with no memory of how and why they came to be intubated.

None of the subjects in the study consented to enrollment beforehand. In fact, the subjects were not even informed that they were part of an experimental protocol until well after they had been forcibly injected, at which point they were told that they could choose to withdraw and have data about them excluded from the study. The investigators justified this by referring to the “waiver of consent” provision of the Common Rule (45 C.F.R. 46.116), which allows consent to be waived if, among other things, the study does not involve more than “minimal risk.”^25^

Yet it is hard to see how the risks of the ketamine study could be considered minimal. Federal regulations state that minimal‐risk studies are those whose anticipated harms or discomforts are no greater than those encountered in daily life or in a routine physical exam. Ketamine is a controlled drug whose possible side effects include hypersalivation, laryngospasm, apnea, dystonia, and vomiting. According to the product labeling for ketamine, about 12 percent of patients experience “emergence reactions,” which vary in severity from pleasant dream‐like states to delirium and hallucinations. Unless the average person is, in the course of their daily life, subjected to controlled drugs with side effects so serious that an injection is likely to result in intubation, the risks of this study were far more than minimal.^26^

It is worth noting that the investigators did not pursue another potential avenue for conducting the study—the “exception from informed consent” requirements for emergency research (under FDA regulations at 21 C.F.R. 50.24). Unlike a waiver of informed consent, the exception permits studies with potential harms or discomforts well above the minimal‐risk threshold. It is intended to test interventions in emergency situations in which a medical condition has made it impossible for a subject to give informed consent and a surrogate decision‐maker is unavailable. It is this provision that permits research on, for instance, hemorrhagic shock or traumatic brain injury. But the bar for being granted an exception from informed consent is very high. For example, subjects must be in “life‐threatening” situations in which available treatments are unproven or unsatisfactory and the experimental intervention holds a prospect of direct benefit.^27^

The Hennepin Healthcare investigators tried to justify the minimal‐risk designation by claiming that they were merely conducting an “observational” study, a term that suggests they were simply observing the effects of the two sedatives without altering who was exposed to them. But this was obviously not the case. For six months of the year, Hennepin Healthcare ambulances carried only ketamine. For the other six months, they carried only haloperidol. Which drug a subject received was thus determined not by the clinical judgment of the paramedic but by the time of year when the subject was treated. So even in situations where there were good medical reasons for paramedics to choose one sedative over another, they were limited to a single drug.

The Hennepin Healthcare investigators clearly understood that ketamine could be risky. In a 2013 article, they stated that standing hospital policy was to use ketamine only for “profound agitation involving imminent risk of injury to patient or provider.”^28^ In another, they reported that 63 percent of profoundly agitated patients administered ketamine in prehospital settings required intubation.^29^ Two of the 135 patients suffered cardiac arrest shortly after receiving ketamine and died. (Reports by the medical examiner in each case ruled that the causes of death were factors other than ketamine.)

Despite the known risks and the disturbing outcome of the first study, Hennepin Healthcare investigators began a second study of ketamine in August 2017.^30^ This study tested ketamine against midazolam, a benzodiazepine often used for procedural sedation and in critical‐care settings that is also sometimes used by paramedics in police encounters.^31^ Once again, the choice of sedative was determined by the period when the subject was seen rather than by the clinical judgment of the paramedic, and once again, subjects were enrolled without their consent. The investigators had planned to enroll approximately 420 subjects over the course of a year, but when the public controversy over the studies erupted in June 2018, Hennepin Healthcare announced that it was suspending the study “due to the concerns that have been expressed.”^32^

Over the next three years, FDA inspections of Hennepin Healthcare revealed a series of serious compliance issues. By virtually any measure, the subjects of the ketamine studies qualified as a vulnerable population: they were disproportionately racial minorities, many of whom had a history of mental illness or chemical dependency, and their cognitive capacities were often impaired by illness, injury, or intoxication. For research on vulnerable populations, federal regulations include additional safeguards to protect the rights and welfare of the subjects. According to the FDA, however, the Hennepin Healthcare IRB approved the studies without determining that any such safeguards were in place.^33^

Equally alarming was the finding that Hennepin Healthcare emergency physicians had conducted other studies without obtaining the consent of the subjects. In 2021, the FDA issued a warning letter to a second Hennepin Healthcare emergency physician who had conducted two comparative studies of sedatives for agitated patients in the emergency department without obtaining informed consent.^34^ According to Public Citizen, the total number of subjects enrolled in Hennepin Healthcare studies without consent exceeded seventeen hundred.^35^

Perhaps the most revealing finding from the FDA inspections concerned the absence of effective research oversight. Not only had the Hennepin Healthcare IRB approved the studies without debate; it had not even given the studies a full review. Instead, it approved the studies by “expedited review,” which is permitted under FDA regulations only for minimal‐risk studies (see 21 C.F.R. 56.110) and is typically conducted only by the IRB chairperson or by one or more IRB members delegated by the chairperson. It appears as if a ketamine injection by a paramedic without the consent of the research subject was seen as such a routine procedure that it did not even require an ethical discussion.

A full accounting of the ways in which the Hennepin Healthcare researchers violated federal research guidelines can be found in Public Citizen's letters to the FDA and the Office for Human Research Protections, as well as the resulting FDA investigations. Yet those letters tell only part of the story. Underlying the violations are a larger series of relationships between Hennepin Healthcare and law enforcement that cast the studies in an even darker light.

## Hennepin Healthcare and Law Enforcement

In May 2019, the *Star Tribune* revealed that Jeffrey Ho, the director of Emergency Medical Services at Hennepin Healthcare, was moonlighting as a part‐time sheriff's deputy.^36^ Later investigations found that he was one of three emergency physicians at Hennepin Healthcare who were licensed law enforcement officers, two of whom—Ho and Paul Nystrom—were involved in the ketamine studies.^37^ For some critics of the studies, this revelation added fuel to the suspicion that the research team was sedating subjects for purposes of law enforcement rather than for their medical well‐being.

This was not the first time that such issues had arisen. In 2015, Minneapolis police officers arrested a man suspected of possessing and selling an illegal drug that they believed he was hiding in his rectal cavity. The police wanted a physician to perform a forced cavity search. When a physician at North Memorial Medical Center refused on ethical grounds, the police took the man to Hennepin County Medical Center, where a physician‐police officer, Paul Nystrom, was on staff. Nystrom sedated the man and searched his rectal cavity, finding 2.9 grams of cocaine. In 2021, the Minnesota Supreme Court overturned the man's conviction for drug possession, ruling that the body cavity search violated the man's constitutional rights to dignity, bodily integrity, and personal privacy.^38^

What made the revelations about Hennepin Healthcare's physician‐police officers even more controversial was a long‐standing financial relationship between Hennepin Healthcare and Axon, the manufacturer of Taser stun guns. According to records obtained by the *Star Tribune*, Hennepin Healthcare received more than $1.1 million in consulting and other fees from Axon between 2005 and 2018.^39^ Axon had funded twenty‐two studies conducted by the hospital's research arm. Three emergency physicians in leadership positions at Hennepin Healthcare had financial or professional relationships with Axon, including Ho, who was the Axon medical director, and the hospital's chief medical officer, William Heegard, who served for at least eight years on Axon's medical advisory board.

At the heart of the Axon controversy was a high‐stakes medical and legal issue: whether Tasers are safe. Tasers are marketed to police departments as “nonlethal” (or “less lethal”) weapons: a weapon less likely to kill a person than conventional weapons, such as firearms.^40^ Tasers are used to incapacitate a person so that the person can be approached safely. Like pepper spray or water cannons, Tasers are deployed to allow police officers to control the behavior of people seen as dangerous or threatening without using methods that could result in more serious harm. Yet Tasers are not free of risk. A 2017 Reuters investigation found that over one thousand people had died after police shocked them with Tasers, often in combination with other forms of force.^41^

When the police use a Taser on a person who subsequently dies, a ruling on the official cause of death is a critical factor in wrongful‐death litigation and official investigations. To show that Tasers are not to blame, Axon often leverages relationships with police officers, medical examiners, and medical specialists.^42^ According to Reuters, Axon commissions research papers to demonstrate that Tasers are not responsible for deaths and injuries; it hires physicians to serve as expert witnesses for the defense in litigation against the company or the police; and it cultivates ties with medical examiners, who decide what to list as a cause of death on official death certificates. Axon also has a history of suing its critics, the threat of which has intimidated many medical examiners into changing their findings.^43^

The result is what the *New York Times* has called an “exoneration network” of scientists, lawyers, physicians, and police‐friendly companies whose expert testimony and research papers are used to absolve police officers of responsibility for fatalities, often after police have used Tasers or physical restraints.^44^ According to the *Times*, experts from the exoneration network are far more likely than independent experts to find that deaths in police custody were due to underlying health conditions rather than actions by the police.

A similar kind of bias has been identified in Axon‐sponsored research. In 2011, for example, a team at the University of California, San Francisco, School of Medicine found that scientific papers funded by or affiliated with Axon (then called Taser International) were eighteen times more likely than independently authored studies to conclude that Tasers were safe.^45^ In studies in which an author was affiliated with Axon, 96 percent of articles concluded that Tasers were either not harmful or unlikely to be harmful. By contrast, among the articles whose authors included no one affiliated with the manufacturer, only 55 percent reached the same conclusions.

Prominent among the Axon‐funded researchers was Hennepin Healthcare's Ho, who became an Axon consultant in 2004 and its contract medical director in 2009.^46^ In 2015‐2016, according to a report by Reuters, Axon paid Hennepin Healthcare nearly $250,000 to help fund Ho's position and reserve a portion of his time for Taser research. Ho's name has appeared on more than one hundred Axon‐funded studies, articles, or presentations, and he has served as an expert in at least two dozen cases in fourteen states. Ho has also spoken out publicly against reports suggesting that multiple Taser shocks can be dangerous. “It has never been scientifically proven that a Taser has directly caused an in‐custody death,” Ho wrote for *Police* magazine in 2005. “The type and magnitude of the electrical charge that the Taser employs makes this association extremely unlikely.”^47^

When the *Star Tribune* reported the financial relationships between Axon and Hennepin Healthcare physicians, some elected officials questioned whether those relationships constituted a conflict of interest.^48^ Yet even if they did constitute a conflict, it was not a hidden one. The Axon‐funded investigators disclosed their funding on their published studies of Tasers, and their ketamine studies were not funded by Axon. According to hospital officials, the relationships between Axon and Hennepin Healthcare physicians did not violate any of its conflict‐of‐interest policies.

Yet the elected officials were still right to be concerned. What is troubling about the relationship between Axon and Hennepin Healthcare is not a straightforward conflict of interest, nor is it simply a clash between the mission of a safety‐net hospital and that of a for‐profit corporation. Rather, it concerns the promotion of a concept far more nebulous and complex: the contested diagnosis of “excited delirium.”

## Excited Delirium

In a 2007 issue of *Police* magazine, Ho published an article titled “How to Respond to Excited Delirium.” Excited delirium, he wrote, is a life‐threatening medical emergency with a high risk of death. It is characterized by a constellation of signs and symptoms including “agitation, incoherence, elevated body temperature, paranoia, inappropriate and often violent behavior, constant motion, and feats of incredible strength.” Police officers need to recognize the condition quickly and call EMS for help because people with excited delirium “will likely need rapid and aggressive medical sedation.”^49^

For two decades, the grave risks of excited delirium have been used to justify the aggressive use of ketamine.^50^ The first case history of ketamine as a “prehospital chemical restraint” was published in 2004 by Ho and a Hennepin Healthcare colleague, John Hick.^51^ Over the next decade or so, emergency physicians at Hennepin County Medical Center and other institutions published a number of articles supporting the use of ketamine, often using the dangers of excited delirium as a justification. Their message: better to sedate people against their will than allow them to risk sudden death. By 2016, a deputy editor of the *Annals of Emergency Medicine* could compare agitated patients to “wild beasts” and write, “Let's take 'em down with a ketamine blow dart.”^52^

This messaging dovetailed with Axon's, which promoted the diagnosis of excited delirium for a different reason: to exonerate Tasers of blame for deaths at the hands of police. The diagnosis was first popularized in 1985, when Charles Wetli, a forensic pathologist, and David Fishbain, a psychiatrist, used it to describe the deaths of seven people intoxicated with cocaine in Miami, five of whom had died in police custody.^53^ According to Wetli and Fishbain, the symptoms of excited delirium include paranoia, fear, panic, physical violence, hyperthermia, unexpected strength, and thrashing, especially after restraints are applied. They suggested the condition could be fatal. Over the next three decades, excited delirium emerged as a popular explanation for deaths in police custody, especially when police were accused of improperly using violence, restraints, chokeholds, or Tasers.^54^

Not everyone was convinced. Critics argued that excited delirium is a questionable diagnosis with no plausible pathophysiological mechanism or biological markers. Nonetheless, the idea of excited delirium steadily gained traction among law enforcement officers and medical examiners, thanks in part to the promotional efforts of Axon. In 2007, Axon purchased and distributed one thousand to fifteen hundred copies of a book called *Excited Delirium Syndrome*, written by Theresa Di Maio, a psychiatric nurse, and her husband, Vincent Di Maio, a forensic pathologist and defense expert for Axon.^55^ As a report by Physicians for Human Rights points out, Axon distributed enough free copies of *Excited Delirium Syndrome* to cover the entire forensic pathology community in the United States.^56^

Another important milestone was the 3rd Annual Sudden Death, Excited Delirium and In‐Custody Death Conference, organized in 2008 by the Institute for the Prevention of In‐Custody Deaths, a corporation cofounded by Michael Brave, the national litigation counsel for Axon, and John Peters, another Axon consultant. IPICD provides training to police officers and offers expert‐witness services. The 2008 conference resulted in a white paper on excited delirium by the American College of Emergency Physicians, which argued that excited delirium is an “acute, potentially life‐threatening medical condition.”^57^ The coauthors included (without disclosures) a number of Axon consultants, including Ho.

Yet when the backlash against excited delirium eventually arrived, it was fierce. A critical turning point came in August 2019, when paramedics in Aurora, Colorado, forcibly administered a large dose of ketamine to Elijah McClain, a twenty‐three‐year‐old Black massage therapist, after diagnosing him with excited delirium.^58^ McClain went into cardiac arrest in the ambulance and was taken off life support four days later. In 2023, the paramedics who sedated him were convicted of criminally negligent homicide.^59^ One was also found guilty of a more serious charge of second‐degree assault for giving a drug without consent or legitimate medical purpose. He received a sentence of five years in prison.

McClain's death did not attract significant national attention in 2019. Things would start to change eight months later, when Minneapolis police officer Derek Chauvin knelt on the neck of George Floyd for over nine minutes while Floyd was handcuffed, restrained, and lying face down in the street. Body‐camera video captured one of the officers worrying that Floyd might have excited delirium.^60^ When Chauvin was tried and convicted of responsibility for Floyd's death two years later, defense attorneys claimed that Minneapolis police had been trained by local physicians to see excited delirium as a dangerous condition that warranted extreme measures.^61^

The deaths of McClain and Floyd marked a massive shift in public opinion about excited delirium. The American Psychiatric Association issued a statement against the diagnosis in 2020, followed by the American Medical Association a year later.^62^ In 2021, the Colorado Department of Public Health and Environment published a report rejecting the diagnosis, stating that it “lends itself to discriminatory practices that result in systemic bias against communities of color.”^63^ The only two professional groups ever to formally recognize the diagnosis, the National Board of Medical Examiners and the American College of Emergency Physicians, both backed away from it in 2023.^64^ Physicians for Human Rights produced an exhaustive report condemning excited delirium, arguing that the diagnosis could not be disentangled from its racist and unscientific origins.^65^

The response in Minneapolis followed a similar trajectory. In January 2022, the Minneapolis Police Department issued a statement saying that police training “no longer uses the term excited delirium.”^66^ However, video material obtained by the *Star Tribune* soon afterwards showed that Hennepin Healthcare physician‐police officer Nystrom was still training police officers how to respond to excited delirium, insisting that “the condition exists.”^67^ Two years later, the state of Minnesota finally settled the issue when Governor Tim Walz signed a law prohibiting training on excited delirium for police officers.^68^

The collapse of excited delirium removed one of the main justifications for the Hennepin ketamine studies—namely, the grave risk to patients of failing to treat a life‐threatening condition. Yet it is important to remember that violent patients can be a legitimate threat to the safety of health care workers. A 2022 survey by the American College of Emergency Physicians found that 45 percent of emergency physicians reported being assaulted in the previous year, and 85 percent thought that violence in the emergency department was getting worse.^69^ Prehospital settings generally have even fewer resources and personnel to deal with violent patients. As long as behavior control is necessary, both health care workers and patients have an interest in efforts to determine how to carry it out safely and effectively. The question is how the research should be conducted. How should we think about clinical research on an intervention carried out by health care workers that involves significant risks but is not aimed primarily at improving the health of a patient?

## Points of Comparison

Two possible points of comparison are helpful. One is clinical research on what are called “chemical restraints.” This is a term typically used to describe sedatives or other psychotropic medication given to control the behavior of patients judged likely to harm themselves or others.^70^ However, as the Hennepin ketamine studies showed, studies of chemical restraints involving paramedics in the field often differ in a critically important way from conventional studies—namely, the involvement of law enforcement. These encounters often look less like an interaction between a patient and a health care worker than a confrontation between a suspect and a police officer. For this reason, a second point of comparison might be research on “nonlethal” or “less lethal” weapons such as Tasers or pepper spray. We will deal with these two points of comparison in turn.

As far as we can determine, the ethics of clinical trials of chemical restraints has received virtually no attention in the medical literature. Clinical trials of chemical restraints are relatively uncommon, and when they are carried out, the chemical restraints in question are generally treated like conventional medical treatments.^71^ The investigators are typically physicians; the research subjects are typically patients in a health care setting, usually a psychiatric unit or an emergency department; and the ethical framework for the studies does not differ from that of a later‐stage clinical trial of a treatment for an ordinary medical condition. The fact that the purpose of chemical restraints is often to protect health care workers rather than promote the health of research subjects is rarely noted as an area of ethical concern.

Yet several issues about trials of chemical restraints are worth noting, as each raises significant ethical concerns. First, the research subjects in most clinical trials of chemical restraints are likely to qualify as members of a vulnerable population. Indeed, it is often the fact that the patients are mentally impaired by illness, injury, or substance abuse that has made their behavior a problem in need of control. The Common Rule (45 C.F.R. 46.111) requires special safeguards for vulnerable populations, as does the Declaration of Helsinki.^72^ Many commentators argue that research on vulnerable populations is justified only when it cannot be carried out on a less vulnerable group.^73^ In published studies of clinical trials of chemical restraints, however, the vulnerability of the research population in question is rarely mentioned. IRBs need to ask whether such studies could be done on a less vulnerable population (for example, a population that is not made up largely of low‐income, marginalized subjects) and in a setting where potential risks can be minimized.

A second, related issue is the difficulty of obtaining informed consent. Most trials of chemical restraints are carried out without the consent of the subjects or any attempt to get consent from a surrogate decision‐maker. Some authors simply ignore the issue, while others note that chemical restraints are routinely used without consent in ordinary clinical practice.^74^ Yet the Declaration of Helsinki states that research subjects “incapable of giving consent must only be included if the research is likely to either personally benefit them or if it entails only minimal risk and minimal burden.” Trials of chemical restraints often fail to meet either of these criteria. Indeed, it is precisely the fact that chemical restraints are so easily deployed for the safety or convenience of health care workers that sets those studies apart from most other clinical trials. IRBs should ask if a study of chemical restraints could be carried out in a setting in which informed consent can be obtained in advance from a surrogate decision‐maker. They should also ask whether the benefits of the experimental restraint are to the subjects themselves or to health care workers.

A third issue with trials of chemical restraints concerns the risks to subjects. In ordinary circumstances, a clinical trial is typically justified by a favorable ratio of benefits to risks. For instance, a phase 2 or 3 trial of a new intervention involving significant risks to subjects may nonetheless be justified by the potential for significant therapeutic benefit. But how should the ratio of risks to benefits be calculated if the benefits are measured largely in terms of the safety of or convenience to health care workers? In such cases, the ethical calculus looks more like that of many phase 1 safety trials of unapproved drugs or that of challenge studies (or “symptom provocation” studies), which may have social value but typically do not promise any therapeutic benefit to subjects. The subjects in these studies are often healthy volunteers who enroll in exchange for financial compensation. For obvious reasons, the acceptable threshold of risk in these studies is typically much lower than it would be in a clinical trial that holds out the possibility of therapeutic benefit.

A second point of comparison for the ketamine trials is research on nonlethal weapons.^75^ This type of research is often much less formalized than research on drugs and devices. To the extent that humans are involved as research subjects for trials of nonlethal weapons, it is usually as healthy volunteers. For instance, Axon sponsored several human studies of Tasers on police officers at sales demonstrations, often in exchange for a free Taser or a case of beer.^76^ While studies of nonlethal weapons at universities may be submitted to an IRB, testing of nonlethal weapons is not formally overseen by any regulatory or oversight body.^77^ In addition, most studies of nonlethal weapons are not clinical trials. Rarely is one type of nonlethal weapon compared to another in the way that ketamine was compared to haloperidol and midazolam at Hennepin Healthcare. If ketamine is being tested as a nonlethal weapon, it is worth asking how such a study differs from conventional medical research.

Perhaps it differs is in its purpose: to study ways of altering a person's behavior, often by harming or disabling them. Physicians clearly possess expertise that could aid in the development of safer nonlethal weapons, but critics have questioned whether their involvement is ethically acceptable. Vivienne Nathanson compares the participation of physicians in weapons research to the participation of physicians in research on torture, enhanced interrogation, or execution. Even if the purpose of such research is to develop interventions that are safer and more humane, she argues, participation by physicians is contrary to the goals of medicine, which are to relieve suffering and improve health.^78^ Her argument has some precedent in the stands taken by professional organizations against the involvement of physicians and nurses in legally authorized executions.^79^

No sign of this controversy appears in published studies of nonlethal weapons. When physicians at Wake Forest University deployed Taser shocks on patients with implanted electrocardiogram sensors, they expressed no ethical reservations about the research.^80^ Nor did physicians at the University of California, San Diego, when they had human subjects inhale pepper spray.^81^ When physicians testing nonlethal weapons on human subjects acknowledge ethical concerns, it is typically about the safety of the weapon, not about whether their professional obligations have been compromised.

In some ways, research on ketamine resembles the problem of dual‐use scientific research, in which the results of the research can have both good and bad applications.^82^ For example, research on anthrax or smallpox may produce scientific knowledge that leads to better medical treatments, but it may also be used to develop biological weapons. With dual‐use research, however, the worry is usually that bad actors will use scientific knowledge in ways that the researchers did not intend. The Hennepin ketamine trials, in contrast, were designed to study the *intended* use of the interventions: how fast they work, how long they work, and what effects and side effects they produce.

Yet even the use of ketamine as intended could lead to unintended consequences. One of the most troubling aspects of chemical restraints is how easily they become substitutes for more humane but less convenient interventions, such as the use of de‐escalation techniques, better staff‐to‐patient ratios, and changes to the treatment environment. Often, it is precisely the effectiveness of the chemical restraints that contributes to their widespread use. For example, when the use of antipsychotics as chemical restraints in U.S. nursing homes skyrocketed in the 2000s, it was partly because the newer atypical antipsychotics were (wrongly) seen as safer than the older drugs.^83^ A similar point has been made about Tasers. Once Tasers became widely available, police began to use them on people for whom they would never have considered using a firearm, often in situations that could have been resolved peacefully.^84^ At least one study has found that Tasers have increased police violence rather than lessened it.^85^

## The Ethics of Behavior Control

It is a platitude of clinical ethics that sound ethical judgments require thick, detailed descriptions. Yet that kind of description is often absent in the ethics of medical research, where judgments are often rendered solely in terms of principles violated and federal guidelines breached. We have tried to show why the ethical problems of Hennepin Healthcare ketamine studies cannot be fully appreciated without an understanding of the larger context in which they took place—namely, the controversial relationship between Hennepin Healthcare and law enforcement, the history of racial discrimination by the Minneapolis police, and the role of the questionable diagnosis of excited delirium in the decision to forcibly sedate a patient.

The larger question raised by the Hennepin Healthcare ketamine studies concerns the ethics of testing interventions aimed at controlling behavior rather than treating a medical condition. The use of chemical restraints has been ethically controversial for at least three decades, yet the ethics of conducting research on chemical restraints has received scarcely any attention. We have suggested that at least some of the published studies of chemical restraints violate federal guidelines regarding consent and the protection of vulnerable populations. More importantly, we have argued that federal guidelines fail to capture all the relevant ethical issues at stake in such research.

## Acknowledgment

This work was supported by a grant from the Robert E. Hopper Family Fund.
